# Mind Blindness

**Published:** 1890-03

**Authors:** 


### Mind Blindness.

Two cases in which this curious symptom was observed have recently been recorded in the *Archiv f. Psych.*, vol. xxi. In the first, recorded by Lissauer, a man of eighty had complained for a month of inability to find his way about, to tell his own position in a room, and to recognize objects, although his perception of light was scarcely impaired. Although he could not recognize objects by looking at them, he at once perceived and named them by means of tactile or auditory impressions from them. On examination he was found to have absolute right homonymous hemianopsia. He had some aphasia, and could not read, but he could write. Perception and discrimination of colors in this case were preserved.

In the second case, recorded by Siemerling, the onset was sudden. At first visual memory only was impaired; but he soon failed to recognize objects, even when he touched, tasted or heard them. On examination he was found to have absolute right homonymous hemianopsia, together with amblyopia in the left field in each eye. Color sense was lost on both sides. There was also amnesic aphasia. In this case very great improvement occurred, the amblyopia on the left side improved, and color vision returned. In neither case was there any change in the fundus. The association of "mind blindness" with hemianopia, and occasionally with loss of color sense, has also been observed by Wilbrand, Charcot, Swanzy and others. It is, however, very rare, while hemianopia is not uncommon; and Siemerling's case, where there was amblyopia in the left field, with complete loss of vision in the right, gives support to the hypothesis of Dr. Gowers that it occurs only when the cortical lesion is double.—*Lancet*, Jan. 25, 1890.

